# Serum Neurofilament Light Chain and Glial Fibrillary Acidic Protein as Biomarkers in Primary Progressive Multiple Sclerosis and Hereditary Spastic Paraplegia Type 4

**DOI:** 10.3390/ijms232113466

**Published:** 2022-11-03

**Authors:** Christoph Kessler, Christoph Ruschil, Ahmed Abdelhak, Carlo Wilke, Aleksandra Maleska, Jens Kuhle, Markus Krumbholz, Markus C. Kowarik, Rebecca Schüle

**Affiliations:** 1 Department of Neurodegenerative Diseases, University of Tübingen, Hoppe-Seyler-Str. 3, 72076 Tübingen, Germany; 2 Hertie Institute for Clinical Brain Research, University of Tübingen, Otfried-Müller-Str. 27, 72076 Tübingen, Germany; 3 German Center for Neurodegenerative Diseases (DZNE), Otfried-Müller-Str. 23, 72076 Tübingen, Germany; 4 Department of Neurology and Stroke, University of Tübingen, Hoppe-Seyler-Str. 3, 72076 Tübingen, Germany; 5 Department of Neurology, Weill Institute for Neurosciences, University of California San Francisco (UCSF), 675 Nelson Rising Lane, San Francisco, CA 94158, USA; 6 Division Translational Genomics of Neurodegenerative Diseases, Hertie-Institute for Clinical Brain Research, University of Tübingen, Otfried-Müller-Str. 27, 72076 Tübingen, Germany; 7 Department of Neurology, University Hospital and University of Basel, Petersgraben 4, 4031 Basel, Switzerland; 8 Multiple Sclerosis Centre and Research Center for Clinical Neuroimmunology and Neuroscience (RC2NB), Departments of Biomedicine and Clinical Research, University Hospital and University of Basel, Spitalstr. 2, 4031 Basel, Switzerland; 9 Department of Neurology and Pain Treatment, Immanuel Klinik Rüdersdorf, University Hospital of the Brandenburg Medical School Theodor Fontane, 15562 Rüdersdorf bei Berlin, Germany; 10 Department of Neurology, Technische Universität München, Ismaninger Str. 22, 81675 Munich, Germany

**Keywords:** PPMS, HSP, SPG4, biomarkers, Serum NfL, Serum GFAP

## Abstract

In patients with slowly progressive spastic paraparesis, the differential diagnosis of primary progressive multiple sclerosis (PPMS) and hereditary spastic paraplegia (HSP) can be challenging. Serum neurofilament light chain (sNfL) and glial fibrillary acidic protein (sGFAP) are promising fluid biomarkers to support the diagnostic workup. Serum NfL is a marker of neuroaxonal decay sensitive to temporal changes, while elevated sGFAP levels may reflect astrocytal involvement in PPMS. We assessed sNfL and sGFAP levels in 25 patients with PPMS, 25 patients with SPG4 (the most common type of HSP) and 60 controls, using the highly sensitive single-molecule array (Simoa) platform. Patients were matched in age, sex, age at onset, disease duration and disease severity. Serum NfL levels were significantly increased in PPMS compared to SPG4 (*p* = 0.041, partial η² = 0.088), and there was a trend toward relatively higher sGFAP levels in PPMS (*p* = 0.097). However, due to overlapping biomarker values in both groups, we did not find sNfL and sGFAP to be useful as differential biomarkers in our cohort. The temporal dynamics indicate sNfL and sGFAP levels are most markedly elevated in PPMS in earlier disease stages, supporting their investigation in this group most in need of a diagnostic biomarker.

## 1. Introduction

Despite recent advances, establishing the correct diagnosis in patients with slowly progressive spastic paraparesis can remain a challenge. After exclusion of macroscopic structural lesions (e.g., cervical/thoracic spinal stenosis, spinal neoplasia, spinal falx meningioma), primary progressive multiple sclerosis (PPMS) and Hereditary Spastic Paraplegia (HSP) often remain as differential diagnoses which are difficult to clinically distinguish. However, the correct diagnosis has significant consequences regarding patient counselling, treatment and overall management. Both PPMS and HSP come with challenges in the diagnostic workup. PPMS is typically diagnosed based on clinically progressive disease, cerebral/spinal T2-hyperintense lesions on MRI and/or oligoclonal bands (OCB) specific to cerebrospinal fluid (CSF) [1]. However, cerebral lesions are usually less frequent than in relapsing–remitting MS (RRMS) [2,3] and therefore can be missed. Furthermore, oligoclonal bands in CSF are not obligatory [4]. The diagnosis of HSP, on the other hand, relies on the detection of pathogenic variants in HSP genes. However, genetic tests fail to detect 50% of cases [5]. Furthermore, the differentiation between both conditions can be further complicated by the presence of unspecific white matter lesions, or those of vascular origin.

Therefore, accurate and easily accessible fluid biomarkers are warranted to facilitate the diagnostic workup of progressive spastic paraplegia, especially at early disease stages. Serum neurofilament light chain (sNfL) and serum glial fibrillary acidic protein (sGFAP) are promising candidates for this task: NfL is a neuronal protein abundantly found in large myelinated axons [6]. Serum NfL is close to being established as a fluid biomarker in some neurological diseases, for instance as a prognostic and especially therapy response biomarker in MS [7]. In SPG4, the most common genetic subtype of HSP, sNfL levels are elevated compared to controls [8], but—as far as results from different studies can be compared—to a lesser extent than in PPMS [9]. GFAP is the major intermediate cytoskeletal protein of astrocytes [10]; serum levels are increased in PPMS [9,11], whereas there are no data on sGFAP levels in HSP. However, the pathobiology of pure HSP with underlying axonal degeneration of the pyramidal tract does not suggest an elevation of sGFAP levels. Although both PPMS and pure HSP are diseases of the central nervous system, the high correlation of CSF and serum levels [9,12] favors measuring them in blood to develop easily accessible fluid biomarkers. Given these promising characteristics, we investigate sNfL and sGFAP as diagnostic biomarkers to differentiate PPMS from HSP.

## 2. Results

The demographic characteristics of PPMS patients, SPG4 patients and controls were well balanced as detailed in Table 1 and described in Section 4. Controls had a median age at serum sampling of 53.0 years (interquartile range 47.5–61.1). The analysis of disease severity yielded a median EDSS score of 3.5/10 (IQR 3.0–6.0) in PPMS patients, and a median SPRS score of 12.0/52 (IQR 10.0–16.5) in SPG4 patients. The median cross-sectional disease progression (points gained per year in EDSS/SPRS divided by disease duration) was 0.5 EDSS points (IQR 0.3–0.8) in PPMS patients and 1.2 SPRS points (IQR 0.9–1.9) in SPG4 patients. All PPMS patients had T2-hyperintense lesions in locations typical for multiple sclerosis on brain MRI scans, and T2-hyperintense spinal cord lesions were found in 88% (21/24) of available spinal MRI scans in PPMS. By contrast, no T2-hyperintense lesions typical for multiple sclerosis were detected in the available brain MRI studies of SPG4 patients, and none of the SPG4 patients had spinal cord lesions on MRI. Oligoclonal bands in CSF were positive in 86% (19/22) of PPMS patients and negative in all SPG4 patients (*n* = 5) with available data. Findings of MRI studies, motor, somatosensory and visual evoked potentials and CSF analysis in PPMS and SPG4 patients are summarized in Table 1 and provided in detail in Supplementary Table S1.

**Figure 1:**
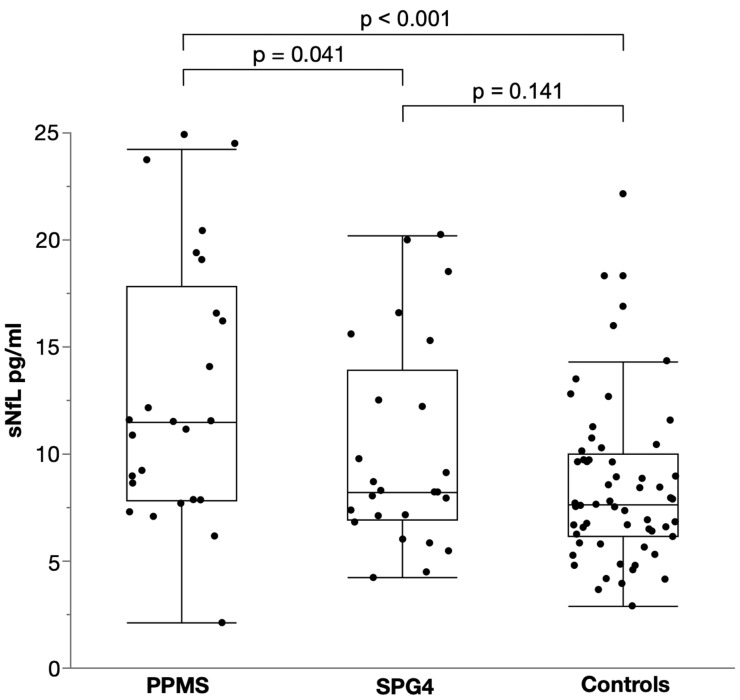
Scatter and box plot of serum NfL levels in PPMS, SPG4 and controls. Horizontal lines represent medians, boxes show interquartile ranges and whiskers extend to the outermost data points within 1.5 interquartile ranges.

Levels of sNfL were significantly elevated in PPMS with a median of 12.8 pg/mL (IQR 7.8–17.8) compared to SPG4 with a median of 8.2 pg/mL (IQR 6.9–13.9; two-way ANCOVA, *p* = 0.041, F (1, 46) = 4.414, B = 0.104, partial η² = 0.088; Figure 1). Controlling for age and sex, this resulted in a 27% elevation of sNfL in PPMS compared to SPG4 as calculated by back-transformation of the log-level coefficient B. Patients with PPMS also had significantly higher levels of sNFL than controls with a median of 7.7 pg/mL (IQR 6.1–10.0), translating into an increase of 43% (two-way ANCOVA, *p* < 0.001, F (1, 81) = 14.508, B = 0.155, partial η² = 0.152). By contrast, sNfL levels in SPG4 patients did not differ from those in controls (two-way ANCOVA, *p* = 0.141, F (1, 81) = 24.008, B = 0.05, partial η² = 0.027).

**Figure 2:**
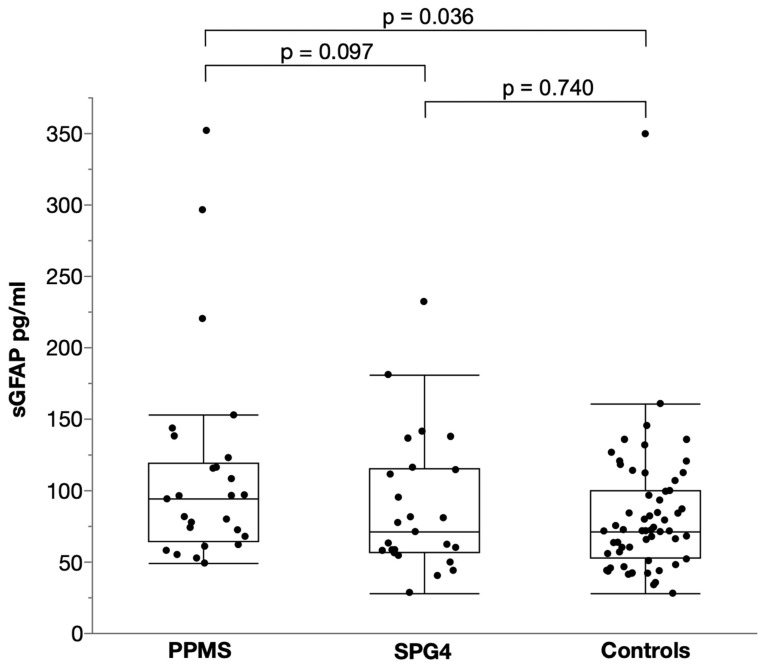
Scatter and box plot of serum GFAP levels in PPMS, SPG4 and controls. Horizontal lines represent medians, boxes show interquartile ranges and whiskers extend to the outermost data points within 1.5 interquartile ranges.

For sGFAP, there was trend towards higher levels in PPMS (median 93.9 pg/mL, IQR 64.7–119.4) than in SPG4 (median 70.9 pg/mL, IQR 56.9–115.1) controlling for age and sex, but this difference did not reach significance (two-way ANCOVA, *p* = 0.097, F (1, 46) = 6.851, B = 0.085, partial η² = 0.059; Figure 2); levels in PPMS were estimated to be 22% higher than in SPG4. Compared to controls (median 71.5 pg/mL, IQR 52.7–99.6), sGFAP levels were significantly elevated in PPMS (two-way ANCOVA, *p* = 0.036, F (1, 81) = 4.531, B = 0.096, partial η² = 0.053) controlling for age and sex, resulting in a 25% increase. They did not differ between SPG4 and controls (two-way ANCOVA, *p* = 0.74, F (1, 81) = 3.832, B = 0.015, partial η² = 0.001).

**Figure 3:**
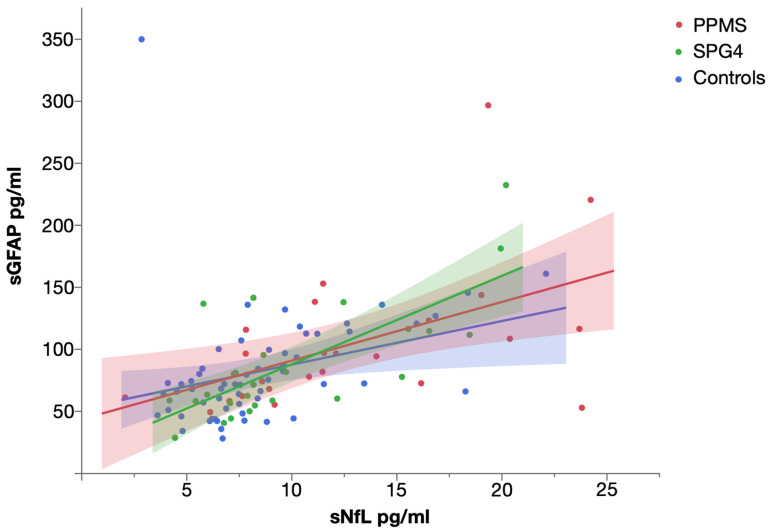
Scatter plot of serum NfL and serum GFAP levels in PPMS, SPG4 and controls, with linear fit. Shaded areas represent 95% confidence intervals.

Levels of sNfL and sGFAP were significantly correlated in PPMS (Spearman’s ρ = 0.563, *p* = 0.003), SPG4 (Spearman’s ρ = 0.630, *p* = 0.001) and controls (Spearman’s ρ = 0.527, *p* < 0.001) (Figure 3).

To explore the usefulness of sNfL and sGFAP as diagnostic biomarkers to differentiate SPG4 and PPMS, we performed a ROC analysis. Both sNfL (AUC 0.64, 95% CI 0.485–0.795, *p* = 0.09) and sGFAP (AUC 0.61, 95% CI 0.452–0.768, *p* = 0.184) could not reliably distinguish PPMS from SPG4. Combining sNfL and sGFAP by using the product of log values as the test variable did not improve the performance (AUC 0.646, 95% CI 0.493–0.803, *p* = 0.076). By contrast, sNfL proved to perform moderately well in differentiating patients with PPMS from controls (AUC = 0.733, 95% CI 0.613–0.852, *p* = 0.001), and sGFAP showed a low, but significant discriminatory power for these groups (AUC = 0.640, 95% CI 0.515–0.765, *p* = 0.043). Patients with SPG4 could not be distinguished from controls by sNfL (AUC 0.589, 95% CI 0.456–0.723, *p* = 0.196) or sGFAP (AUC 0.519, 95% CI 0.377–0.660, *p* = 0.787).

**Figure 4:**
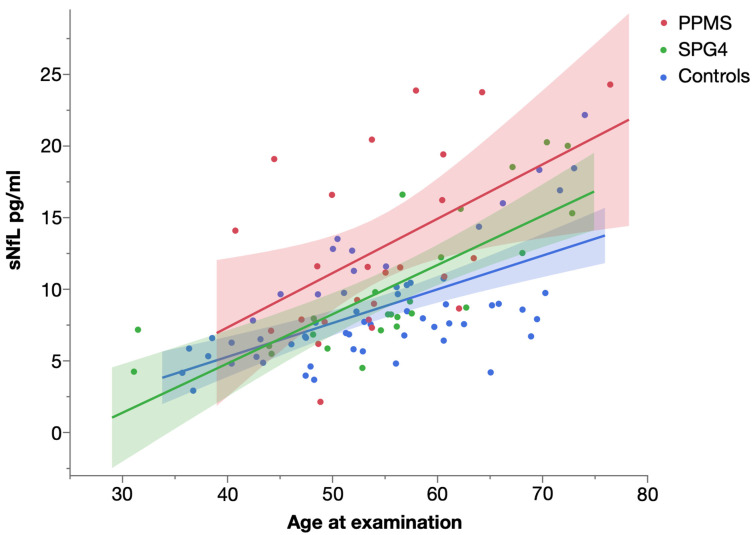
Scatter plot of serum NfL levels in PPMS, SPG4 and controls by age, with linear fit. Shaded areas represent 95% confidence intervals.

Levels of sNfL significantly increased with age in all three groups (Figure 4). The strongest influence was found in SPG4 (Spearman’s ρ = 0.872, *p* < 0.001), followed by controls (Spearman’s ρ = 0.585, *p* < 0.001) and patients with PPMS (Spearman’s ρ = 0.439, *p* = 0.028).

**Figure 5:**
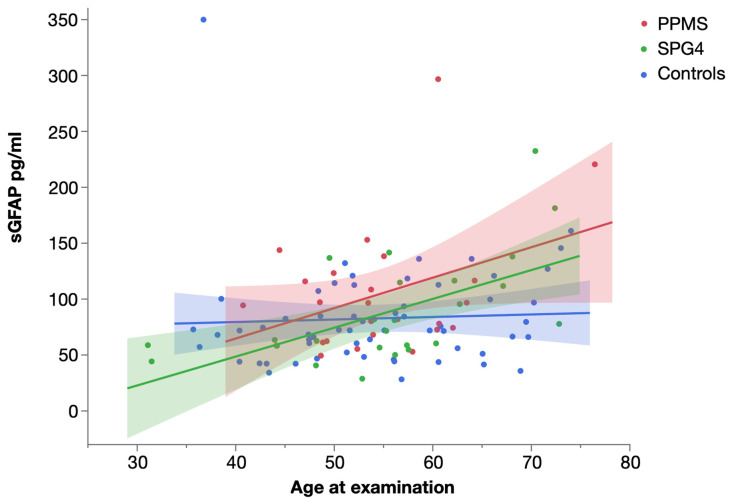
Scatter plot of serum GFAP levels in PPMS, SPG4 and controls by age, with linear fit. Shaded areas represent 95% confidence intervals.

A statistically significant correlation of sGFAP levels and age (Figure 5) was only seen in SPG4 patients (Spearman’s ρ = 0.554, *p* = 0.004), whereas it was absent in PPMS (possibly not significant due to the higher variability; Spearman’s ρ = 0.198, *p* = 0.344) and controls (Spearman’s ρ = 0.213, *p* = 0.103).

**Figure 6:**
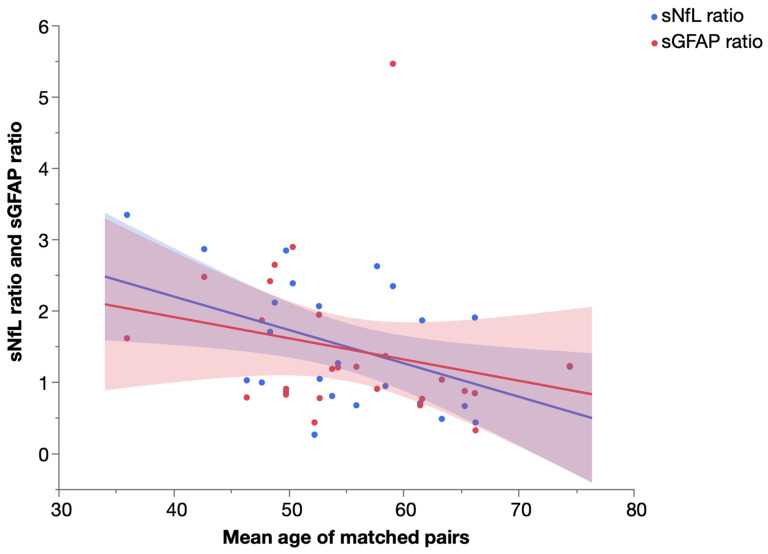
Ratio of serum NfL and serum GFAP levels in matched pairs of PPMS and SPG4 patients by mean age, with linear fit. Shaded areas represent 95% confidence intervals.

To further assess the temporal dynamics of sNfL and sGFAP, we performed a pair-matching procedure of SPG4 and PPMS patients as detailed in the Section 4.4 and calculated the ratio (biomarker level_PPMS patient_: biomarker level_SPG4 patient,_ e.g., 20 pg/mL: 10 pg/mL = 2) of sNfL and sGFAP levels for each matched pair. We then analyzed the correlation of the sNfL and sGFAP ratio and the mean age of matched pairs (Figure 6). While we found a significant increase in sNfL levels in PPMS compared to SPG4 (see above), the sNfL ratio of PPMS and SPG4 patients significantly declined in older subjects (Spearman’s ρ = −0.410, *p* = 0.042). The same trend was observed for sGFAP, but without reaching significance (Spearman’s ρ = −0.370, *p* = 0.069).

In regard to sex, median sGFAP levels were higher in females than in males in all groups (Table 2). However, this difference did not reach significance in any group (Mann-Whitney test, PPMS: *p* = 0.103, SPG4: *p* = 0.744, controls: *p* = 0.100). Levels of sNfL did also not significantly vary between males and females (Mann-Whitney test, PPMS: *p* = 0.624, SPG4: *p* = 0.744, controls: *p* = 0.625), with relative differences being smaller for sNfL than for sGFAP.

When assessing the influence of disease duration and disease severity on sNfL and sGFAP levels, we controlled for age by calculating the individual sNfL and sGFAP ratio for each patient compared to a control matched in age and sex as detailed in the Section 4.4. This ratio expresses the fold increase of sNfL and sGFAP levels in an individual patient compared to a matched control. In SPG4, we found a significant correlation of the sNfL ratio and disease duration (Spearman’s ρ = 0.432, *p* = 0.031), whereas disease severity as measured by the SPRS score did not correlate with the sNfL ratio (Spearman’s ρ = 0.164, *p* = 0.433). The same applied to the sGFAP ratio (disease duration: Spearman’s ρ = 0.295, *p* = 0.153, disease severity: Spearman’s ρ = 0.131, *p* = 0.531) in SPG4. In PPMS, the sNfL ratio was not correlated with disease duration (Spearman’s ρ = 0.245, *p* = 0.237) or disease severity as measured by the EDSS score (Spearman’s ρ = 0.289, *p* = 0.161). There was also no significant correlation of the sGFAP ratio and disease duration (Spearman’s ρ = 0.187, *p* = 0.371) or disease severity (Spearman’s ρ = 0.281, *p* = 0.173) in PPMS. Cross-sectional disease progression did not correlate with the sNfL ratio (PPMS: Spearman’s ρ = −0.148, *p* = 0.481; SPG4: Spearman’s ρ = −0.117, *p* = 0.578) or the sGFAP ratio (PPMS: Spearman’s ρ = −0.066, *p* = 0.753; SPG4: Spearman’s ρ = 0.118, *p* = 0.573).

We did not find a significant difference in sNfL levels (Mann-Whitney test, *p* = 0.935) and sGFAP levels (Mann-Whitney test, *p* = 0.531) between PPMS patients previously treated with GCS and those without previous treatment. Age (Mann-Whitney test, *p* = 0.807) and sex (two-sided chi-squared test, *p* = 0.428) did not differ between both subgroups.

The explorative analysis of the influence of spinal cord lesions in PPMS on sNfL and sGFAP levels did not indicate a considerable effect. Both patients with (*n* = 21) and without (*n* = 3) spinal cord lesions on MRI had a median sNfL level of 11.5 pg/mL (with spinal cord lesions: range 2.1–24.3 pg/mL; without spinal cord lesions: range 8.9–19.1 pg/mL). The median sGFAP level of patients with spinal cord lesions was 93.9 pg/mL (range 48.9–296.2 pg/mL), patients without spinal cord lesions had a median sGFAP level of 96.7 pg/mL (range 67.6–143.3 pg/mL). *p* values were not calculated due to the small sample size.

Serum NfL and GFAP levels of PPMS patients by disease activity (T1 gadolinium-enhancing or new/enlarging T2 lesions on MRI scan) are displayed in Supplementary Figure S1. *P* values were not calculated due to the small sample size. It should be noted that MRI data were usually acquired before the date of serum sampling (see Supplementary Table S1 for details), so the correlation of fluid biomarker levels and MRI data should be evaluated cautiously.

## 3. Discussion

In this study, we provide the first direct comparison of sNfL and sGFAP levels in PPMS and SPG4, two diseases sharing the clinical hallmark of slowly progressive spastic paraparesis. While we found significantly higher sNfL levels and a trend toward higher sGFAP levels in PPMS compared to SPG4, neither of these two biomarkers could reliably differentiate PPMS from SPG4 on a single subject level, possibly due to the notable overlap of biomarker values. Therefore, our results do not support the use of sNfL or sGFAP as a diagnostic biomarker in patients with slowly progressive spastic paraparesis to distinguish between PPMS and HSP, but may help to assess the contribution of neuroinflammation and neurodegeneration in PPMS. A marked elevation of sGFAP levels could be expected in PPMS compared to SPG4 given the major role of aberrant astrocyte activation in the pathophysiology of PPMS [13,14]. On the contrary, no activation of astrocytes has been described in cellular models of SPG4 [15,16]. This is in line with our finding that sGFAP levels do not differ between patients with SPG4 and controls. Remarkably, the elevation of sGFAP levels in PPMS compared to SPG4 did not reach significance, while sNfL levels were significantly increased in PPMS. In addition, the estimated elevation was slightly higher for sNfL (27%) than for sGFAP (22%). This also applied to the comparison of PPMS and controls (sNfL: 43%, sGFAP: 25%). The lack of a pronounced increase in sGFAP levels in PPMS compared to SPG4 might be attributed to the small sample size (25/25 subjects), as the difference between PPMS patients and controls (25/60 subjects) who had sGFAP levels similar to SPG4 was significant.

On the one hand, sNfL levels in PPMS appear to be more markedly elevated than sGFAP levels when assessing the magnitude of the increase in patients compared to controls [11,17]. On the other hand, sGFAP levels are higher in progressive than in relapsing-remitting MS (PPMS [11], SPMS [18] and PPMS/SPMS [12,19]). This seeming paradox hints at a dual pathobiology of progressive disease in PPMS consisting of compartmentalized neuroinflammation and pronounced neurodegenerative processes. While elevated sGFAP levels probably reflect the involvement of astrocytes in continuing neuroinflammation [9,20,21], increased sNfL levels are attributable to neuroaxonal degeneration [12,22]. Analyzing the temporal dynamics of sNfL and sGFAP indicated a decreasing difference between PPMS and SPG4 patients with age as the sNfL ratio of matched pairs declined significantly in older subjects, and the same non-significant trend was observed for sGFAP. This suggests that the elevation of sNfL and sGFAP levels in PPMS compared to SPG4 is most pronounced in early stages of disease and lessens with age, which is in line with histopathological findings supporting dying-out of inflammatory and neurodegenerative pathology with age [23].

For NfL, similar temporal dynamics have been described in a larger SPG4 cohort and other slowly progressive neurodegenerative diseases in comparison to controls [8,24,25,26]. This phenomenon is presumably caused by a progressive loss of axons which are the source of NfL release, leading to a lower increase of sNfL levels at later stages [8]. Since the lower release of NfL and subsequent lower increase of sNfL levels in progressive disease is a mechanism independent of the underlying pathobiology, it may also occur in PPMS and explain the vanishing difference in sNfL levels compared to SPG4.

For sGFAP, the trend toward a lower increase in later stages might be related to a decreasing role of neuroinflammation in progressive disease, as a shift to neurodegeneration as a driver of disease progression has been proposed in PPMS [27]. Although this has been challenged by studies showing persistent neuroinflammation [23] and the effectiveness of Ocrelizumab in PPMS [28], the significantly better response to ocrelizumab of patients with gadolinium-enhancing lesions on baseline MRI [29] may point to a predominance of neurodegeneration in patients without inflammatory activity on MRI, possibly concurring with lower sGFAP levels.

Analyzing the influence of age on levels of sNfL and sGFAP in PPMS and SPG4 further hints to different temporal dynamics, as sNfL and sGFAP levels were significantly correlated with age in SPG4, while the correlation of sNfL levels and age was lower in PPMS, and the correlation of sGFAP levels and age was not significant in PPMS. The concurrence of biomarkers and age indicates a comparatively steady rise in sNfL and sGFAP levels in SPG4, while this correlation is missing in PPMS. However, the missing correlation in PPMS may also be caused by the relatively high variability of biomarker levels in PPMS as illustrated by the wide confidence intervals in Figure 4 and Figure 5.

A limitation of our study is the relatively small number of PPMS and SPG4 patients compared to previous studies investigating either PPMS or SPG4. The small size might impair the adjustment of sNfL and sGFAP levels for demographic factors (e.g., age and sex), as some statistical analyses used to control for these factors require larger sample sizes. Another limitation is the transferability of findings to other subtypes of HSP, as our study comprised a cohort of SPG4 patients to represent those with a pure HSP phenotype and our results may not be directly applicable to other genotypes. However, our findings on biomarker levels in SPG4 might be representative for the whole group of HSP patients with a pure phenotype, as (i) SPG4 is by far the most frequent genotype within this group and (ii) genotypes with similar clinical patterns can be expected to show similar biomarker levels due to a consistent extent of affected fiber tracts and rate of neuroaxonal decay [8,30].

## 4. Subjects and Methods

### 4.1. Subjects

We recruited 25 patients with PPMS, 25 patients with SPG4 and 60 controls from the Department of Neurology, Hertie Institute for Clinical Brain Research, University Hospital Tübingen. Progressive spastic paraparesis was the first and main sign in all patients. The diagnosis of PPMS was based on established criteria [1]. Of the 25 patients with PPMS, 15 (60%) were treatment-naïve, and 10 (40%) had received intravenous corticosteroids, however not within 3 months prior to serum sampling. None of the patients had received any other disease-modifying treatment. In the SPG4 cohort, the diagnosis was genetically confirmed in all patients. We selected an SPG4 cohort to represent patients with HSP, as SPG4 typically leads to a pure HSP phenotype mimicking PPMS and is the most frequent HSP genotype, accounting for up to 60% of autosomal-dominant and 30% of all HSP cases [5,31,32,33]. None of the patient samples were included in previous publications. The control cohort comprised 40 healthy individuals and 20 individuals who underwent blood sampling for diagnostic purposes but did not have any neurological deficits at examination. These subjects were diagnosed with depression (*n* = 6), primary headache disorder (*n* = 5), restless legs syndrome (*n* = 4), functional disorders (*n* = 4) and trigeminal autonomic cephalgia (*n* = 1), none of which is known to alter levels of sNfL or sGFAP. Age was equally distributed across PPMS, SPG4 and control cohorts (Kruskal-Wallis test, *p* = 0.669). There were also no differences in sex between the cohorts (male/female ratio 12:13 in PPMS, 13:12 in SPG4, 32:28 in controls; two-sided chi-squared test, *p* = 0.904). Age at onset (Mann-Whitney test, *p* = 0.763) and disease duration (Mann-Whitney test, *p* = 0.123) did not differ significantly between patients with PPMS and SPG4. Median values and interquartile ranges of the aforementioned data are detailed in Table 1. For correlation analyses, disease severity was measured using the Expanded Disability Status Scale (EDSS) for PPMS and the Spastic Paraplegia Rating Scale (SPRS) for SPG4, respectively. To assess disease severity across patients with PPMS and SPG4, we established three subgroups according to the ambulation status (free ambulation/walking aid required/ loss of ambulation). We did not find a significant difference in ambulation status between patients with PPMS and SPG4 (PPMS: 17/6/2 patients, SPG4: 18/7/0 patients; two-sided chi-squared test, *p* = 0.349). Magnetic resonance imaging, evoked potentials and CSF analysis were usually performed before the date of serum sampling as detailed in Supplementary Table S1. Written informed consent was obtained from all participants or their legal representatives. The local ethics committee approved of the study (172/2018BO2, 199/ 2011BO1).

### 4.2. Biomaterial

Serum samples were frozen at −80 °C within 60 min after collection, stored in the local biobank of the Hertie Institute for Clinical Brain Research, shipped on dry ice to the Department of Neurology, University Hospital Basel, Switzerland and analyzed without previous thaw-freeze cycles.

### 4.3. Serum NfL and GFAP Measurements

Serum GFAP concentrations were measured in duplicate with the ultrasensitive Simoa technology (Quanterix, Billerica, MA, USA) using the singleplex Simoa GFAP Discovery Kit (Quanterix) on the HD-X analyzer according to manufacturer’s instructions. The concentrations of sNfL were measured in duplicates by the same technology as previously described [34]. The concentrations of all samples were higher than the concentration of the lowest calibrator reaching acceptance criteria, and lower than the concentration of the highest calibrator reaching acceptance criteria. Each run contained three native serum reference samples with mean sNfL levels of 5.5 pg/mL, 17.4 pg/mL and 32.2 pg/mL and mean sGFAP levels of 54.2 pg/mL, 59.3 pg/mL and 78.6 pg/mL. Inter-assay CV as calculated from the reference samples were 4.9%, 0.7% and 6.6% for sNfL and 5.1%, 6.1% and 12.7% for sGFAP. Individual intra-assay CV as calculated from patient and control samples (*n* = 110) yielded a median of 4.5% (IQR 2.3–6.8%) for sNfL and 3.5% (IQR 1.4–6.2%) for sGFAP.

### 4.4. Statistical Analysis

To compare sNfL and sGFAP levels in patients with PPMS and SPG4 and controls, we performed an analysis of covariance (ANCOVA) for every combination of the aforementioned groups. The ANCOVA included age as a covariate and sex as a cofactor, therefore controlling for the influence of these parameters. In order to compare the temporal dynamics of sNfL and sGFAP levels in patients with PPMS and SPG4, we pair-matched each PPMS patient to a SPG4 patient, using age and sex as matching criteria (propensity score matching in the mode “optimal matching” with a caliper of 0.2, according to Thoemmes, F. (2012). Propensity score matching in SPSS. arXiv:1201.6385). We then analyzed the correlation of the matched pairs’ sNfL and sGFAP ratio and their mean age.

We used a similar approach when assessing the correlation of biomarker levels with disease severity and duration in order to control for the influence of age and sex, since the assumptions of ANCOVA were not consistently met due to smaller sample size. Therefore, we matched all PPMS and SPG4 patients to a control, using age and sex as matching criteria as described above. We then calculated the ratio of sNfL and sGFAP levels for the matched pairs. This ratio was used for non-parametric correlation analyses, as it is expected to reflect the age- and sex-adjusted fold increase of sNfL and sGFAP in each patient. The use of sNfL and sGFAP as diagnostic biomarkers was assessed by ROC analyses. All statistical analyses were performed using IBM SPSS Statistics for Macintosh Version 28.0.1.1 (IBM Corp., Armonk, NY, USA). Figures were created using JMP Version 16.0.0 (SAS Institute Inc., Cary, NC, USA) and Affinity Designer Version 1.8.4 (Serif (Europe) Ltd., Nottingham, UK).

## 5. Conclusions

In conclusion, our study could not establish sNfL and sGFAP as diagnostic biomarkers to differentiate between PPMS and SPG4, the most common type of hereditary spastic paraplegia. However, our comparison of matched PPMS and SPG4 cohorts provides a fluid biomarker perspective to the pathobiology of PPMS. This comparison suggests that inflammatory and neurodegenerative pathology in PPMS decrease with age, as the temporal dynamics of sNfL and sGFAP show an elevation of each biomarker in PPMS compared to SPG4, which is most pronounced in younger patients and lessens with age. Considering diagnostic biomarkers, the greater elevation of sNfL and sGFAP levels in younger PPMS patients warrants prospective longitudinal studies investigating levels shortly after disease onset, as the discriminatory power is possibly higher in this group of patients most in need of a diagnostic biomarker.

## Figures and Tables

**Table 1:** Demographic data, MRI, evoked potentials and cerebrospinal fluid analysis in PPMS and SPG4 patients.

		PPMS	SPG4
Age at serum sampling (years)		53.8 (48.8–60.5)	56.2 (48.9–62.5)
Age at onset (years)		47.0 (35.5–53.0)	47.0 (39.0–51.0)
Disease duration (years)		7.7 (3.8–13.4)	11.2 (7.3–15.5)
MRI (T2-hyperintense lesions)	periventricular	25/25	3/19 (consistent with cerebral microangiopathy)
	juxtacortical	24/25	
	infratentorial	19/25	1/19 (consistent with pontine microangiopathy)
	spinal	21/24	0/17
CSF	Pleocytosis (>5 cells per μL)	6/19	0/9
	Abnormal IgG Index (>0.7)	7/12	0/6
	Positive OCB	19/22	0/5
Abnormal MEP	Upper limbs	9/12	0/17
	Lower limbs	13/15	10/18
Abnormal SEP	Upper limbs	5/7	0/4
	Lower limbs	13/14	6/10
Abnormal VEP		12/15	0/3

X/Y, X number of patients with abnormal findings, Y number of all patients with available data (e.g., spinal MRI was available in 24 PPMS patients and abnormal in 21 of those patients); MRI, magnetic resonance imaging; CSF, cerebrospinal fluid; OCB, oligoclonal bands; MEP, motor evoked potentials; SEP, somatosensory evoked potentials; VEP, visual evoked potentials. Values of age at serum sampling, age at onset and disease duration are detailed as medians and interquartile ranges.

**Table 2:** Levels of sNfL and sGFAP in PPMS, SPG4 and controls.

		PPMS	SPG4	Controls
Total	sNfL	12.8 pg/mL (7.8–17.8)	8.2 pg/mL (6.9–13.9)	7.7 pg/mL (6.1–10.0)
	sGFAP	93.9 pg/mL (64.7–119.4)	70.9 pg/mL (56.9–115.1)	71.5 pg/mL (52.7–99.6)
Men	sNfL	10.3 pg/mL (7.7–18.3)	8.2 pg/mL (7.6–11.0)	7.6 pg/mL (6.4–10.0)
	sGFAP	76.8 pg/mL (58.5–110.5)	70.9 pg/mL (58.2–88.2)	64.5 pg/mL (43.6–98.5)
Women	sNfL	11.5 pg/mL (8.8–18.0)	7.7 pg/mL (6.2–18.0)	7.90 pg/mL (5.3–10.2)
	sGFAP	96.7 pg/mL (75.7–145.2)	87.1 pg/mL (54.7–131.2)	76.5 pg/mL (66.4–109.0)

Values are detailed as medians and interquartile ranges.

## Data Availability

The data presented in this study are available on reasonable request and as patient consent allows from the corresponding author.

## Supplementary Materials

The following supporting information can be downloaded at: https://www.mdpi.com/article/10.3390/ijms232113466/s1, Figure S1: Levels of serum NfL (A) and serum GFAP (B) of PPMS patients by disease activity on cerebral MRI (T1 gadolinium-enhancing or new/enlarging T2 lesions); Table S1: Detailed demographic data, MRI, evoked potentials and cerebrospinal fluid analysis in PPMS and SPG4 patients.
