# Supplementary figures and images for: Serum Neurofilament Light Chain and Glial Fibrillary Acidic Protein as Biomarkers in Primary Progressive Multiple Sclerosis and Hereditary Spastic Paraplegia Type 4
Source: Int J Mol Sci. 2022 Nov 3;23(21):13466. doi: 10.3390/ijms232113466 (PMC9657281; doi:10.3390/ijms232113466)

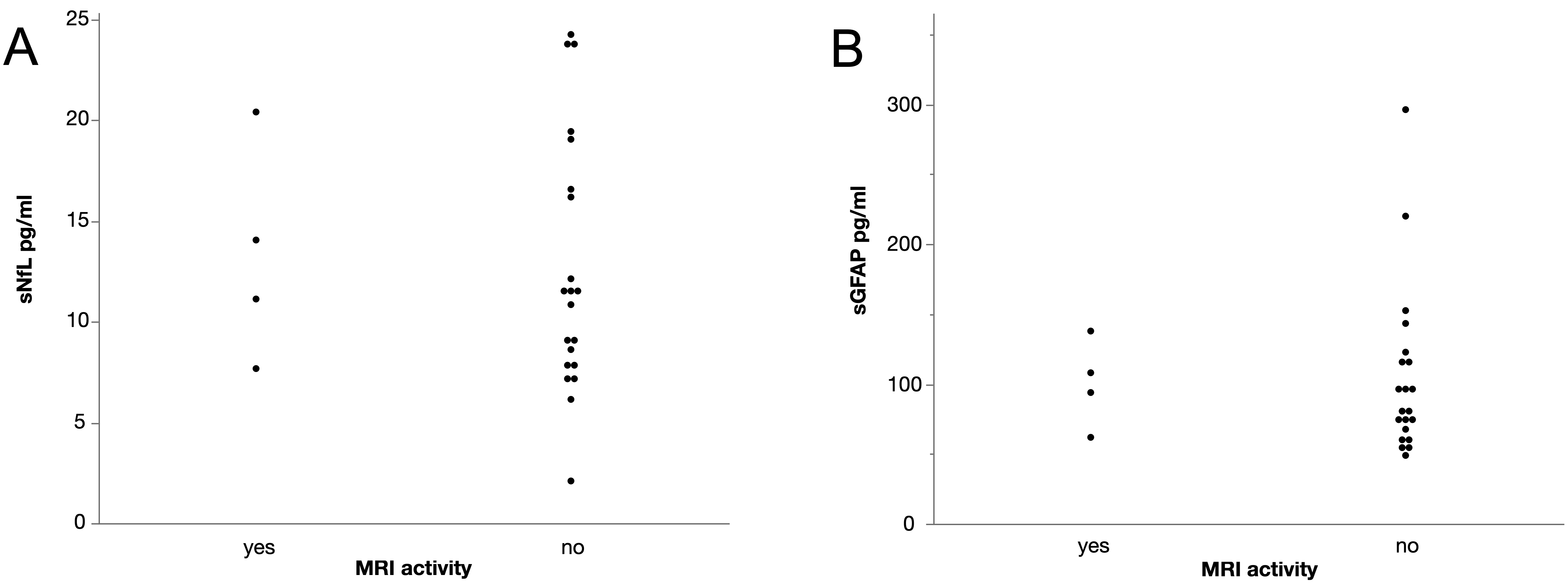

Supplement: Supplementary file 1 [file ijms-23-13466-s001.zip › Supplementary Figure S1.png]
